# Rapid Alleviation of Parkinson’s Disease Symptoms via Electrostimulation of Intrinsic Auricular Muscle Zones

**DOI:** 10.3389/fnhum.2017.00338

**Published:** 2017-06-28

**Authors:** Yusuf O. Cakmak, Hülya Apaydin, Güneş Kiziltan, Ayşegül Gündüz, Burak Ozsoy, Selim Olcer, Hakan Urey, Ozgur O. Cakmak, Yasemin G. Ozdemir, Sibel Ertan

**Affiliations:** ^1^Department of Anatomy, School of Medical Sciences, Otago UniversityDunedin, New Zealand; ^2^Department of Neurology, Cerrahpasa School of Medicine, Istanbul UniversityIstanbul, Turkey; ^3^Global Dynamic Systems (GDS) ARGE, Teknopark IstanbulIstanbul, Turkey; ^4^Department of Electrical Engineering, College of Engineering, Koç UniversityIstanbul, Turkey; ^5^Department of Neurology, School of Medicine, Koç UniversityIstanbul, Turkey

**Keywords:** Parkinson’s disease, electrostimulation, auricular muscles, PPN, STN, mesencephalon, locomotor, neuromodulation

## Abstract

**Background:** Deep brain stimulation of the subthalamic nucleus (STN-DBS) and the pedunculopontine nucleus (PPN) significantly improve cardinal motor symptoms and postural instability and gait difficulty, respectively, in Parkinson’s disease (PD).

**Objective and Hypothesis:** Intrinsic auricular muscle zones (IAMZs) allow the potential to simultaneously stimulate the C2 spinal nerve, the trigeminal nerve, the facial nerve, and sympathetic and parasympathetic nerves in addition to providing muscle feedback and control areas including the STN, the PPN and mesencephalic locomotor regions. Our aim was to observe the clinical responses to IAMZ stimulation in PD patients.

**Method:** Unilateral stimulation of an IAMZ, which includes muscle fibers for proprioception, the facial nerve, and C2, trigeminal and autonomic nerve fibers, at 130 Hz was performed in a placebo- and sham-controlled, double-blinded, within design, two-armed study of 24 PD patients.

**Results:** The results of the first arm (10 patients) of the present study demonstrated a substantial improvement in Unified Parkinson’s Disease Ratings Scale (UPDRS) motor scores due to 10 min of IAMZ electrostimulation (*p* = 0.0003, power: 0.99) compared to the placebo control (*p* = 0.130). A moderate to large clinical difference in the improvement in UPDRS motor scores was observed in the IAMZ electrostimulation group. The results of the second arm (14 patients) demonstrated significant improvements with dry needling (*p* = 0.011) and electrostimulation of the IAMZ (*p* < 0.001) but not with sham electrostimulation (*p* = 0.748). In addition, there was a significantly greater improvement in UPDRS motor scores in the IAMZ electrostimulation group compared to the IAMZ dry needling group (*p* < 0.001) and the sham electrostimulation (*p* < 0.001) groups. The improvement in UPDRS motor scores of the IAMZ electrostimulation group (ΔUPDRS = 5.29) reached moderate to high clinical significance, which was not the case for the dry needling group (ΔUPDRS = 1.54). In addition, both arms of the study demonstrated bilateral improvements in motor symptoms in response to unilateral IAMZ electrostimulation.

**Conclusion:** The present study is the first demonstration of a potential role of IAMZ electrical stimulation in improving the clinical motor symptoms of PD patients in the short term.

## Introduction

**FIGURE 1:**
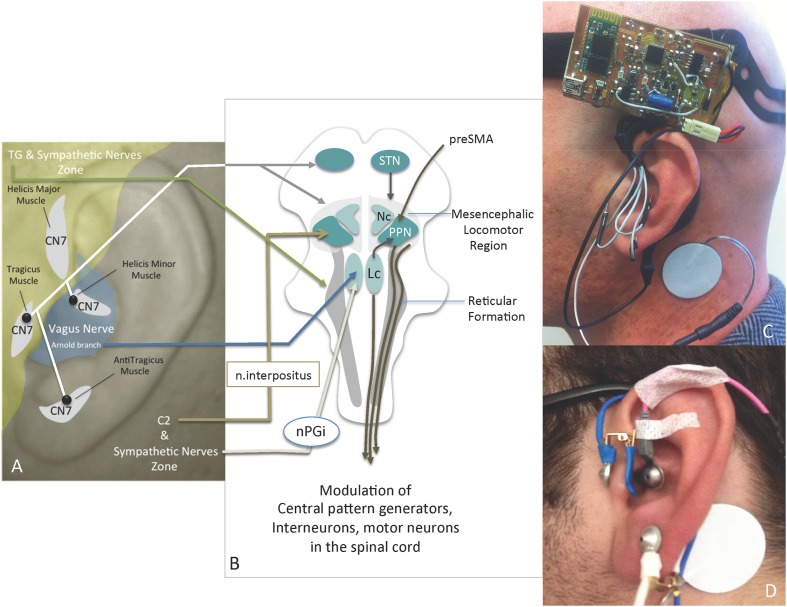
**(A)** Innervation of the ear, the IAMZs and IAMZ electrode locations (black dots); **(B)** relevant anatomical pathways of IAMZs via auricular nerves for motion regulation and mesencephalic locomotor regions (modified from Burn, 2013); **(C)** active stimulation with needle electrodes of the IAMZ and with TENS electrode of the SCM; **(D)** placebo group TENS electrode placements in the IAMZ and SCM; CN7, facial nerve; C2, C2 spinal nerve; TG, trigeminal nerve; Nc, nucleus cuneatus; Lc, locus coerulei; nPGi, nucleus paragigantocellularis; STN, subthalamic nucleus; PPN, pedunculopontine nucleus. *Yellow zone*: trigeminal nerve + sympathetic nerve zone; *Gray zone*: C2 + sympathetic nerve zone; *White areas*: intrinsic auricular muscles zones. *Blue area*: Arnold branch of the vagus nerve, C2, CN7, and sympathetic nerves overlapping zone.

Bilateral high-frequency stimulation of the subthalamic nucleus (STN), commonly known as deep brain stimulation of the STN (STN-DBS), provides a significant improvement in cardinal motor symptoms and in the control of drug-induced complications of Parkinson’s disease (PD). To be clinically effective, stimulation of the STN must be applied at frequencies greater than 100 Hz (Limousin et al., 1995; Limousin et al., 1997), and electrostimulation is usually delivered at 130 Hz (Little and Brown, 2012). Effective stimulation was shown to be associated with a significant decrease in the activity of the ipsilateral primary sensorimotor cortex at rest and a significant increase in premotor, anterior cingulate, pre-supplementary motor areas (pre-SMAs) and dorsolateral prefrontal cortices during movement (Payoux et al., 2004). On the other hand, axial symptoms, such postural instability and gait difficulty (PIGD), freezing of gait, and impaired speech, have been reported to be resistant to STN-DBS and levodopa treatments (Hamani et al., 2007; Tattersall et al., 2014). Recent clinical results have indicated the potential effectiveness of deep brain stimulation of the pedunculopontine nucleus (PPN-DBS) on PGID (Hamani et al., 2007; Tattersall et al., 2014). It has been demonstrated that lesions of the PPN induce gait deficits (Karachi et al., 2010; Tattersall et al., 2014). Moreover, cholinergic cell loss in the caudal PPN is accompanied by PIGD (Hirsch et al., 1987; Zweig et al., 1989; Karachi et al., 2010; Tattersall et al., 2014). Therefore, PIGD is thought to be related to the mesencephalic locomotor region rather than dopaminergic motor centers such as the STN (Garcia-Rill, 1991). The major components of the mesencephalic locomotor region are the PPN and the cuneiform nucleus (Garcia-Rill, 1991). In the context of different outcomes of STN-DBS and PPN-DBS, PPN dysfunction likely contributes to PIGD in PD (Tykocki et al., 2011). The PPN receives direct inputs from the pre-SMA and the basal ganglion to modulate muscle tone by directly exciting pontine reticular formation (Burn, 2013). Central pattern generators in the spinal cord can also be modulated directly and indirectly by the PPN (**Figure 1B**). Notably, sensory proprioceptive afferents have also been demonstrated to modify these patterns (Jankowska et al., 1983; Takakusaki et al., 2008; Burn, 2013).

Although altered proprioception is not a prominent symptom of PD, proprioception deficits have been reported in PD patients (Lee et al., 2013), and improvements in proprioception have been reported with STN stimulation (Lee et al., 2013). Selective muscle afferent nerve stimulation has been reported to cause significant activation in motor-related areas compared with cutaneous stimuli (Wardman et al., 2014). Muscle afferent stimulation evokes more widespread cortical, subcortical, and cerebellar activations than cutaneous afferents (Wardman et al., 2014). Separate precentral and post-central excitation foci were observed with muscle afferent stimulation, emphasizing the importance of muscle afferent nerve stimulation in the modulation of cortical motor areas (Wardman et al., 2014). Therefore, peripheral nerve stimulation may also hold the potential as an alternative and minimally invasive approach to modulate the activity in the premotor, anterior cingulate, pre-SMA and dorsolateral prefrontal cortices and basal ganglia such as the STN and the PPN and should be investigated in PD. Median nerve stimulation has been shown to result in the activation of STN neurons, which form a motor homunculus (Nambu et al., 1996, 1997, 2000; Hanajima et al., 2004). In theory, stimulation of other peripheral motor nerves may also activate motor homunculus of the STN.

Upper facial muscles around the orbital region are controlled bilaterally by the motor cortex through the facial nerve (Mima et al., 1999). In addition, unilateral STN-DBS has been demonstrated to induce strictly contralateral motor-evoked potentials in the trapezius, deltoid, biceps, and thenar muscles; however, the same stimulus reportedly always induces bilateral motor-evoked potentials in the orbicularis oculi, orbicularis oris, masseter, and sternocleidomastoid in Parkinson’s patients (Costa et al., 2007). The intrinsic auricular muscles of the tragicus and antitragicus muscles have also been shown to simultaneously contract with the orbicularis oculi muscles (Matsuo and Hirose, 1987). The latter demonstration is also indirect proof of bilateral cortical connections of intrinsic auricular muscles, such as in the orbicularis oculi.

In the context of these anatomical relationships, unilateral stimulation of the intrinsic auricular muscle zones (IAMZs) may hold potential for bilateral feedback stimulation of muscle feedback and motor driver cortical areas. In addition to the possible proprioceptive outcomes of IAMZ stimulation, contributions of the facial nerve branches stimulating the intrinsic auricular muscles (Fujita, 1934; Matsuo and Hirose, 1987), the vagus nerve via the Arnold branch as a parasympathetic contribution (Alvord and Farmer, 1997; Peuker and Filler, 2002), the trigeminal nerve via the auriculotemporal nerve branch (Alvord and Farmer, 1997; Peuker and Filler, 2002) and the C2 spinal nerve within the great auricular nerve (Alvord and Farmer, 1997; Peuker and Filler, 2002) to IAMZ have been demonstrated in the literature (**Figure 1A**). In addition, cervical sympathetic nerves are distributed to the auricula within the ear arteries (Daniel and Paton, 1975; Lambru et al., 2013) (**Figure 1A**). Therefore, stimulation of the IAMZ has the potential for synchronous stimulation of the C2 spinal nerve, the trigeminal nerve, and sympathetic and parasympathetic nerves (**Figure 1A**), each of which may contribute to motor regulation.

It has been demonstrated that the C2 spinal nerve forms anatomical connections with the deep cerebellar nucleus of the interpositus (represented by the nucleus globosus and the emboliformis in humans) in animals (Matsushita and Xiong, 2001). The interpositus has projections to the PPN and from the PPN to the STN (Schell and Strick, 1984; Hazrati and Parent, 1992; Lavoie and Parent, 1994; Harting, 1997; McFarland and Haber, 2000; Muthusamy et al., 2007). In addition, the C2-interpositus nucleus-PPN-reticulospinal axis may play a cardinal role in motion and position regulation: a substantial body of evidence suggests that the reticulospinal system is essential and plays a fundamental role in the integration of commands for whole-body movement and postural adjustment rather than the movement of a single limb (Magni and Willis, 1964; Keizer and Kuypers, 1984, 1989; Drew and Rossignol, 1990; Luccarini et al., 1990; Drew, 1991; Massion, 1992; Mori et al., 1992; Kably and Drew, 1998; Schepens and Drew, 2003) (**Figure 1B**). Of note, the reticulospinal system is also directly excited by the PPN and mediates DBS-PPN to modulate muscle tone in PD patients (Burn, 2013) (**Figure 1B**). In addition to the potential effects of the C2 spinal nerve on the reticulospinal system via the interpositus nucleus-PPN axis, the mesencephalic trigeminal nucleus as the principal nucleus of the facial muscles’ proprioceptive center, projects to the reticular formation and to the spinal cord via propriospinal neurons (Lawrence and Kuypers, 1968; Gorska and Sybirska, 1980; Alstermark et al., 1981, 1989; Hobbs et al., 1992; Usunoff et al., 1997; Whishaw et al., 1998; Sasaki, 2004; Jankowska and Edgley, 2006) (**Figures 1A,B**). This connection also underlines the potential role of intrinsic auricular muscle proprioception feedback on reticular formation and the possible influences on movement and posture, in addition to C2-related pathways, on reticular formation and propriospinal neurons. The C2 spinal nerve projects to the lower ear lobe and the corresponding skin that overlays the intrinsic muscles of the antitragicus muscle by the greater auricular nerve and the trigeminal nerve; its auriculotemporal branch innervates the skin areas over the tragicus and helicis major muscles (Matsuo and Hirose, 1987; Alvord and Farmer, 1997; Peuker and Filler, 2002; Lambru et al., 2013; Drake et al., 2015) (**Figure 1A**).

In addition to the possible indirect autonomic effects of IAMZ stimulation via the C2 nerve, autonomic fibers of the auricula also directly contribute to these zones. While the sympathetic nerves overlay the IAMZ within the arterial walls, the Arnold branch of the vagus nerve is distributed only over the helicis minor muscle zone (Matsuo and Hirose, 1987; Alvord and Farmer, 1997; Peuker and Filler, 2002; Lambru et al., 2013; Drake et al., 2015) (**Figure 1A**). Thus far, no neuromodulation studies of the Arnold branch or neurotraces of the efferent connections of the Arnold branch have considered the existence of the helicis minor muscle in the concha area where the Arnold branch is distributed.

The earliest accumulation of α-synuclein and Lewy bodies have been shown to occur in the dorsal motor nucleus of the vagus nerve in PD (Braak et al., 2003). A recent study also showed that chronic impairment of vagus nerve function leads to inhibition of dopamine and that low frequency stimulation of the vagus nerve significantly inhibited the dopamine system in rat brain structures (Ziomber et al., 2012). It is worth noting that low- and high-frequency electrostimulation induce opposite outcomes on the autonomic nerve system and neurotransmitters (Cakmak et al., 2008, 2016; Zhao, 2008), so that high frequency of stimulation may result with the stimulation of the dopaminergic system.

In the context of the demonstrated structural and functional anatomical connections of the IAMZ and the underlined mechanisms of deep brain stimulation studies in PD, the IAMZ is the only potential auricular zone to synchronously stimulate the C2 spinal nerve, the trigeminal nerve, autonomic nerve fibers and proprioceptive centers to modulate motor modulatory centers including mesencephalic locomotor region. We hypothesized that the stimulation of IAMZ would be beneficial to alleviate the PD motor symptoms. A clinical trial is designed to investigate the potential clinical outcomes (motor symptoms) of the IAMZ stimulation in PD patients. We stimulated the IAMZ (which includes the helicis minor, tragicus, and antitragicus muscles) unilaterally (ipsilateral to the dominant PD symptoms) at a high frequency and analyzed the efficacy of this technique on 24 PD patients via a double-blind, placebo- and sham controlled within-subject design, two-armed study.

## Materials and Methods

All subjects provided written informed consent to undergo the procedure. The study was approved by the Ethics Committee of the Koç University, Turkey, and it was carried out in accordance with the Ethical Principles for Medical Research Involving Human (Declaration of Helsinki).

Koç University Clinical Trials Ethics Committee Approval Number: 2015.091.IRB1.018,
ClinicalTrials.gov Identifier: NCT02722824,
Turkey Ministry of Health: Follow up number - 133235.

### Research Participants

Ten volunteer patients with idiopathic PD who had been followed in our movement disorders outpatient clinic were enrolled for the first arm of the study. Another 14 volunteer patients with idiopathic PD with the same criteria were enrolled for the second arm of the study. PD diagnoses were made by a neurologist who was an expert in movement disorders using the UK Parkinson’s Disease Society Brain Bank clinical diagnostic criteria (Hughes et al., 1992).

All patients underwent a detailed neurological examination, and parkinsonian features were rated according to the Unified Parkinson’s Disease Rating Scale (UPDRS) Part III. Patients with a disease duration longer than 2 years and Hoehn and Yahr stage ≥ 2 were included. Patients with cognitive impairment that might prevent cooperation during tests and patients with any other neurological or systemic disease in which electrostimulation was contraindicated were excluded.

### Electrostimulation and Analysis of Symptoms

**FIGURE 2:**
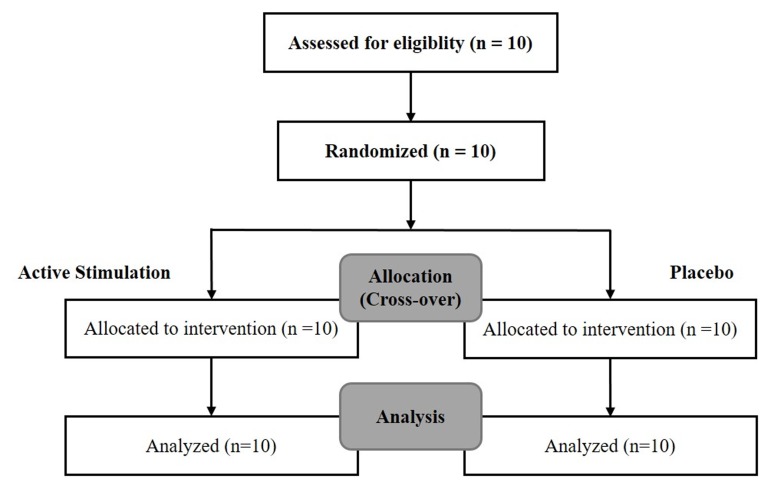
The consort flow chart of the first arm of the study.

*Arm 1* [*10 patients – 10 min of electrostimulation of the IAMZ with needle electrodes and 10 min of placebo using transcutaneous electrodes (TENS) for IAMZ*]*:* two movement disorder specialists blind to the nature of stimulation rated all patients twice according to the motor section of the UPDRS just before the stimulation (baseline evaluation) and 10 min after the onset of stimulation or placebo application. The patients and two movement disorder specialists were informed that there would be two modes of stimulation, sub-threshold stimulation with TENS of the IAMZ (placebo group, **Figure 1D**) and above the sensory threshold with needle electrodes of the IAMZ (active group, **Figure 1C**). In the placebo-control application in which there was no stimulation and the device was in off-mode, the patients and two movement disorder specialists were informed that a new device would be placed on the ear to improve their motor symptoms and that they would not sense the stimulation of the device because of the sub-threshold stimulation of the IAMZ via the TENS. In the active group, the patients and two movement disorder specialists were informed that the stimulation would be above the sensory threshold and applied via the needle electrodes to the IAMZ. In both groups, there was a TENS electrode on the sternocleidomastoid muscle (SCM) (**Figures 1C,D**). The consort flow chart (**Figure 2**) demonstrates the study design in the first arm.

*Arm 2* [*14 patients – 20 min of stimulation in three sessions: electrostimulation of the IAMZ with needles, needling of the IAMZ without electrostimulation (dry needling) and sham region electrostimulation with needles*]: the main aims of the second arm of the study were to assess the following:

- a potential effect of dry needling on the IAMZ without electrostimulation (dry needling of the IAMZ zones without electrostimulation but keeping the needle electrodes connected with to the electrostimulator),
- the potential effect of stimulation of only the C2/sympathetic nerve by ensuring no or minimal stimulation of the SCM, the vagus facial nerve and the trigeminal nerve (sham stimulation),
- the specificity of the IAMZ zone electrostimulation (active group-IAMZ electrostimulation),
- the sole specificity of 130 Hz stimulation (sham stimulation zone),
- a potential long-term effect of the stimulation (50th-minute).

In the second arm of the study, the stimulation duration proceeded to the 20th-minute, and patients were assessed at the 50th-minute (30 min after the termination of 20-min stimulation). The two movement disorder specialists who participated in the first arm and who were blind to the nature of stimulation rated all (*n* = 24) patients twice according to the motor section of the UPDRS.

**FIGURE 3:**
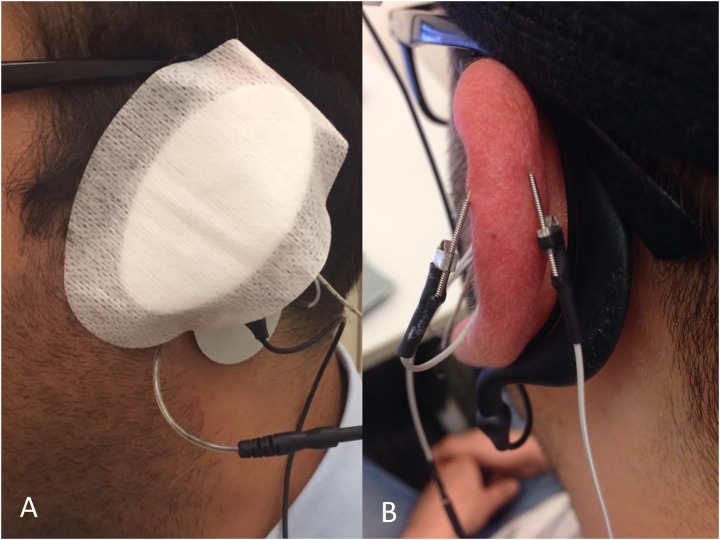
**(A)** A pad covered the ear to ensure observer blindness in the groups of the second arm of the study. **(B)** Sham stimulation zone needle electrode placement.

**FIGURE 4:**
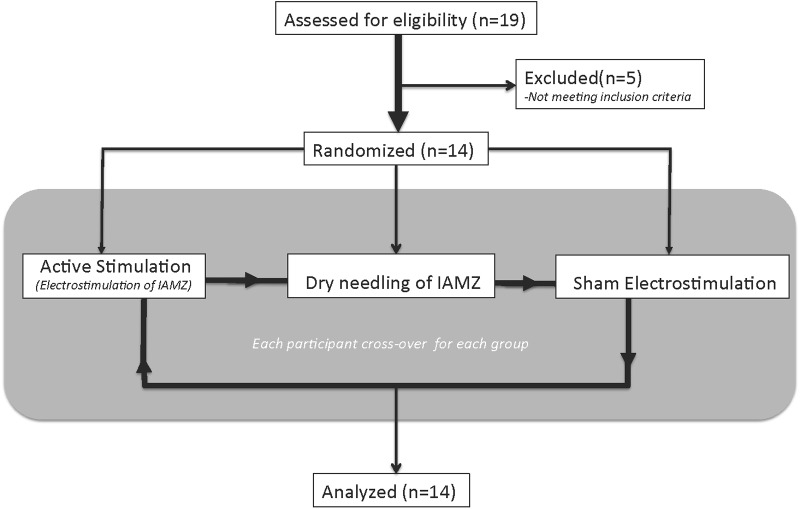
The consort flow chart of the second arm of the study.

The two movement disorder specialists were blinded to the type of stimulation by covering the entire auricula with cotton pads, including the needle electrodes (**Figure 3A**). Patients were also informed that there would be three modes of stimulation: sub-threshold stimulation with needles (dry needling of the IAMZ without electrostimulation but with the needles connected to stimulator as in the needle electrostimulation group, **Figure 1C**) and electrostimulation with needle electrodes above the sensory threshold at two different auricular zones (electrostimulation with needle electrodes on the IAMZ **Figure 1C** and a sham region **Figure 3B**). These groups ensured that the patients were blind to the active stimulation group. A part of the upper helix that is innervated by C2/sympathetic nerves but free or with minimal contributions of the SCM, vagal, facial, and trigeminal nerves was selected as the sham electrostimulation region (**Figure 3B**). The consort flow chart in **Figure 4** demonstrates the study flow in the second arm.

### Stimulation Procedure

All examinations and electrostimulations were performed during “off” periods of the patients (when the effect of the dopaminergic drug was minimal). We designed and built an electrical signal generator with a changeable voltage output (0–5 V), frequency (2–300 Hz) and pulse length (50–200 μs) with external electrodes able to be connected to press needles (KINGLI, China) placed on the three intrinsic auricular muscles (tragicus, antitragicus, and helicis minor muscles). While the needle electrodes were placed on the IAMZ (**Figure 1C**) in the active stimulation and dry needling groups and over the upper helix in the sham stimulation group (**Figure 3B**), the transcutaneous electrode was placed on the SCM 1–2 cm to its mastoid origin (**Figure 1C**) near the electrical stimulation circuit. In the sham group, the transcutaneous electrode was placed but remained inactive to keep the electrical field of stimulation in the selected helix region for C2/sympathetic nerve stimulation. It was also placed and kept inactive in the dry needling group to maintain observer blindness.

The SCM was chosen for surface electrode placement for three reasons:

(1) STN contribution: bilateral SCM activation has been reported during unilateral STN stimulation, which underlines the bilateral STN connections to each SCM (Costa et al., 2007).
(2) Nucleus cuneatus contribution: the mesencephalic locomotor region has two major components: the PPN and the nucleus cuneatus. SCM proprioception projects to the nucleus cuneatus as the neck muscle proprioception center [6] and as a mesencephalic locomotor component (Garcia-Rill, 1991) (**Figure 1B**).
(3) C2 spinal nerve contribution: the skin over the SCM (1–2 cm to its mastoid origin) is also innervated by the C2 spinal nerve (Matsuo and Hirose, 1987; Alvord and Farmer, 1997; Peuker and Filler, 2002; Lambru et al., 2013; Drake et al., 2015) (**Figure 1A**).

In the present study, we used a biphasic wave-form and a stimulation style that alternated the anode and cathode for each stimulus during the stimulation period such that the needle electrodes on the intrinsic auricular muscles and the surface electrode on the SCM served as both the anode and cathode in an alternating manner. Given the much larger contact surface of the transcutaneous electrode compared with the needle electrodes and taking into account the difference in the skin conductance between the needle electrode and the transcutaneous electrode in addition to the low power of the stimulus, the transcutaneous electrode on the SCM would have a minimal impact on the stimulation-related outcomes.

Auricular electrical stimulation was applied with the following STN-DBS parameters: 130 Hz with a wavelength of 100 μs and an intensity just under the pain threshold (1–4 V). The stimulation was administered unilaterally (ipsilateral to the side of prominent symptoms). We did not set a power (V) for the stimulation because such an approach does not eliminate the probability of current differences due to skin conductance differences in different subjects or within subjects during different sessions. To overcome this possible problem, the electrostimulator contained a built-in current-control feature so that the current was stabilized in the range of 100–130 μÅ during the stimulation period by modulating the power and ensuring that the tingling sensation was under the pain threshold. This approach overcomes the interindividual and intraindividual (in active and control sessions) differences in skin conductance.

In addition to the traditional approach of taking into account the dominant symptom side scores of lateralized subitems (items 20–26) for the UPDRS Part-III motor scale scoring, the scores of lateralized subitems on both sides (ipsilateral and contralateral to the dominant symptoms and to the stimulator) were also documented to reveal any potential effects of unilateral stimulation over the bilateral motor symptoms. Moreover, subscores of the UPDRS Part-III were also classified and analyzed as Tremor (items 20–21), Rigidity (item 22), Bradykinesia (items 23–26,31), Gait and Postural Stability (items 27–30) and Bulbar Anomalies (items 18–19), as in previous studies (Postuma et al., 2008). All statistical analyses were performed using Student’s paired *t*-tests (Prism 7 demo version, GraphPad Software, Inc., La Jolla, CA, United States, 2016). In addition, the Bonferroni correction was also applied as a conservative approach for the subscore group analysis [*n* = 2 (active and placebo), *p* < 0.025 in Arm 1, and *n* = 3 (active, dry needling, and sham), *p* < 0.016 in Arm 2]. The second arm of the study, which included three groups, also analyzed via analysis of variance (ANOVA) for group comparisons.

## Results

### Arm 1

The mean age of the patients was 55.7 ±SD 7.8 years, and only one patient was female. The mean disease duration was 8.3 ±SD 4.4 years. Detailed clinical features of the patients are presented in **Table 1**.

**Table 1:** Detailed clinical features of the patients in the first arm of the study.

	Patient 1	Patient 2	Patient 3	Patient 4	Patient 5	Patient 6	Patient 7	Patient 8	Patient 9	Patient 10
Age (years)	61	46	55	48	54	48	61	71	52	61
Gender	F	M	M	M	M	M	M	M	M	M
Dominant hand	R	R	R	R	R	R	R	R	R	R
Affected side at onset	Right	Left	Right	Right	Right	Left	Left	Right	Right	Right
Symptoms at onset	UE BK, R > Tr	UE BK, R	UE BK > Tr	UE and LE BK	UE BK, R > Tr	LE BK	UE and LE Tr	UE Tr	UE BK	UE Tr
PD type	Akinetic rigidity	Akinetic rigidity	Akinetic rigidity	Akinetic rigidity	Akinetic rigidity	Akinetic rigidity	Tremor dominant	Tremor dominant	Akinetic rigidity	Tremor dominant
PD duration (years)	12	4	8	12	6	8	6	17	2	8
Wearing off	+	+	+	+	+	+	+	+	-	+
Dyskinesia	+	+	+	+	-	+	-	+	-	+
Medication	LDED: 1100 mg/day, Amantadine	LDED: 500 mg/day, Amantadine	LDED: 700 mg/day, Amantadine	LDED: 600 mg/day, Amantadine	LDED: 650 mg/day, Amantadine, Rasagiline	LDED: 800 mg/day, Rasagiline	LDED: 1025 mg/day	LDED: 950 mg/day, Amantadine	LDED: 450 mg/day, Rasagiline	LDED: 1100 mg/day, Amantadine
H&Y stage	3	3	2	2	2	2	2	2	2	3

*F, female; M, male; R, right; L, left; UE, upper extremity; LE, lower extremity; BK, bradykinesia; R, rigidity; Tr, tremor; PD, Parkinson’s disease; LDED, levodopa equivalent dose; H&Y, Hoehn and Yahr.*

There were no statistically significant differences between the baseline UPDRS motor scores of the patients in the active and placebo groups (*p* = 0.735). The UPDRS motor scores showed a statistically significant improvement 10 min after auricular stimulation compared with baseline UPDRS scores (*p* = 0.0003, power: 0.99). The placebo group did not show statistically significant differences in UPDRS motor scores compared with baseline (*p* = 0.130). At the 10th-minute, the mean improvement rates were 35% (ΔUPDRS = 5.9) in the active group [mean UPDRS motor scores: 17.0 at baseline, 11.1 at the 10th-minute, standard deviation (SD): 3.6 at baseline and 4.6 at the 10th-minute, ΔUPDRS range = 1.5–12] and 5% (ΔUPDRS = 1) in the placebo-control group (mean UPDRS motor scores: 17.4 at baseline, 16.4 at the 10th-minute, *SD*: 4.6 at baseline and 4.5 at the 10th-minute, ΔUPDRS range = -1.5–4.5).

**FIGURE 5:**
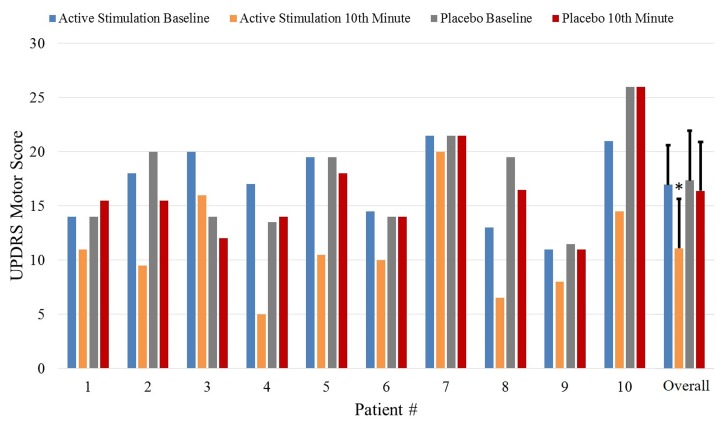
The UPDRS part III scores at baseline and at the 10th-minute for the placebo and active groups and overall comparisons of the first arm. ^∗^Statistically significant.

**FIGURE 6:**
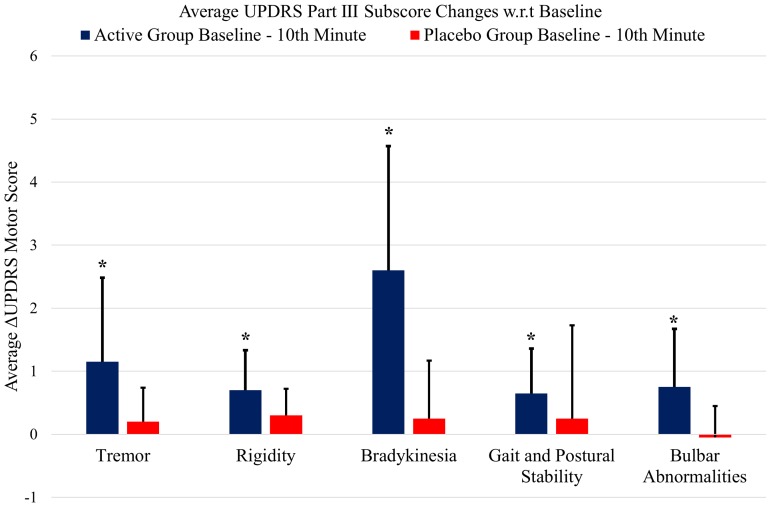
The classified UPDRS subscore differences for group comparisons of the first arm (*p* < 0.05, *t*-test). ^∗^Statistically significant.

**FIGURE 7:**
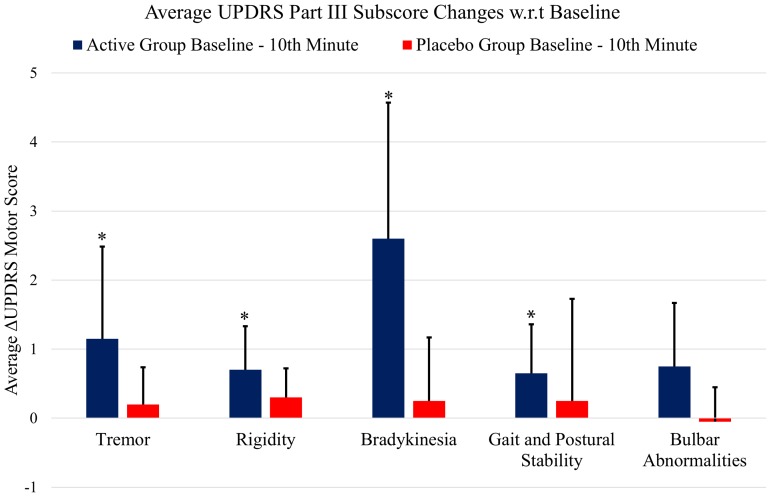
The classified UPDRS subscore differences for group comparisons with Bonferroni correction. ^∗^Statistically significant.

**Figure 5** shows the UPDRS Part-III scores and the group comparison for each case and an overall summary of the first arm. **Figure 6** summarizes the classified UPDRS subscore differences for the group comparisons with *t*-tests, **Figure 7** gives these differences for the group comparisons after the Bonferroni correction. **Table 2** summarizes the *p*-values for the *t*-tests and after the Bonferroni correction of the classified UPDRS subscores for the 10th-minute compared with baseline.

**Table 2:** The *p*-values after Bonferroni correction of the classified UPDRS subscores for the 10th-minute compared with baseline of the first arm study groups.

Subgroup	*p*-Value active group	*p*-Value placebo group
Tremor	***0.023***	0.398
Rigidity	***0.007***	0.051
Bradykinesia	***0.002***	0.413
Gait and postural instability	***0.018***	0.605
Bulbar abnormalities	*0.030*	0.758

**t*-test; *p* < 0.05, Bonferroni correction *p* < 0.025, Italic: significant in *t*-test, bold: significant after Bonferroni correction, bold and italic: significant in *t*-test and Bonferroni.*

The bilateral assessments of the symptomatic improvements of PD patients before and after electrical stimulation revealed that unilateral stimulation of the intrinsic auricular muscles can relieve PD symptoms on both sides at the 10th-minute. The 10th-minute mean improvements in the lateralized (UPDRS Part III subscore 20–26) UPDRS motor subscores were 3.0 on the contralateral side and 3.2 on the ipsilateral side in the active group. The 10th-minute difference in the lateralized UPDRS motor subscores in the placebo-control group were 0.5 on the contralateral side and 0.8 on the ipsilateral side.

### Arm 2

The mean age of the patients was 58.1 ±SD 9.8 years, and 6 of the patients were female. The mean disease duration was 7.9 ±SD 3.7 years. Detailed clinical features of the patients are presented in **Table 3**. Baseline UPDRS motor scores of the three groups were compared via one-way repeated measures ANOVA. There were no statistically significant differences between the baseline UPDRS motor scores of the patients in the active (IAMZ electrostimulation), IAMZ dry needling and sham electrostimulation groups prior to the interventions (pairwise comparison *p*-values: active vs. dry needling: 0.286, active vs. sham: 0.052, sham vs. dry needling: 1.0).

**Table 3:** Detailed clinical features of the patients in the second arm of the study.

	Patient 1	Patient 2	Patient 3	Patient 4	Patient 5	Patient 6	Patient 7	Patient 8	Patient 9	Patient 10	Patient 11	Patient 12	Patient 13	Patient 14
Age (years)	56	47	63	58	54	70	64	45	71	45	63	72	45	60
Gender	M	F	M	F	M	F	M	F	M	F	F	M	M	M
Dominant hand	R	R	R	R	R	R	R	R	R	R	R	R	L	R
Affected side at onset	Right	Right	Right	Right	Right	Right	Left	Left	Right	Left	Right	Left	Right	Right
Symptoms at onset	UE BK	UE TR > BK	UE TR	UE TR > BK	UE TR	UE BK	UE BK	UE TR	UE BK	LE TR	LE TR	BK	TR	BK
PD type	Akinetic rigidity	Akinetic rigidity	Akinetic rigidity	Tremor dominant	Akinetic rigidity	Akinetic rigidity	Akinetic rigidity	Tremor dominant	Akinetic rigidity	Tremor dominant	Tremor dominant	Akinetic rigidity	Akinetic rigidity	Akinetic rigidity
PD duration (years)	7	10	8	15	9	8	1	4	5	8	4	13	8	10
Wearing off	+	+	-	+	+	+	-	-	-	-	-	-	+	-
Dyskinesia	-	+	+	-	+	+	-	-	-	-	+	+	-	+
Medication	LDED: 950 mg/day, Amantadine	LDED: 1200 mg/day, Amantadine	LDED: 300 mg/day, Amantadine	LDED: 1400 mg/day, Amantadine, Rasagiline	LDED: 1316.2 mg/day	LDED: 1000 mg/day	LDED: 500 mg/day	LDED: 940 mg/day, Rasagline	LDED: 660 mg/day	LDED: 840 mg/day	LDED: 800 mg/day	LDED: 850 mg/day, Amantadine	LDED:600 mg/day, Rasagline	LDED: 1162.5 mg/day
H&Y stage	2	2	2	2	3	3	2	2	2	2	2	2	2	2

*F, female; M, male; R, right; L, left; UE, upper extremity; LE, lower extremity; BK, bradykinesia; R, rigidity; Tr, tremor; PD, Parkinson’s disease; LDED, levodopa equivalent dose; H&Y, Hoehn and Yahr.*

In the active group, the UPDRS motor scores were significantly improved at the 50th-minute after the initiation of IAMZ electrostimulation (i.e., 30 min after the termination of the 20-min stimulation) compared with baseline UPDRS scores (*p <* 0.001, power: 0.99). The IAMZ dry needling group also showed statistically significant differences in UPDRS motor scores after the dry needling procedure compared with baseline (*p* = 0.011). However, the improvement in UPDRS motor scores of dry needling stimulation of the IAMZ (ΔUPDRS = 1.54) did not reach clinical significance (Shulman et al., 2010) as electrostimulation of the IAMZ (ΔUPDRS = 5.29). The improvement in the UPDRS score observed in the active group (electrostimulation of the IAMZ) was in the range of moderate to high clinical significance (Shulman et al., 2010). There was no significant difference in UPDRS motor scores in the sham electrostimulation group (*p* = 0.748).

The mean improvement rates at the 50th-minute were 31% (ΔUPDRS = 5.29) in the active group (mean UPDRS motor scores: 17.71 at baseline, 12.42 at the 50th-minute, *SD*: 4.27 at baseline and 4.61 at the 50th-minute, ΔUPDRS range = 0.50–9.50), 9% (ΔUPDRS = 1.54) in the dry needling group (mean UPDRS motor scores: 16.04 at baseline, 14.50 at the 50th-minute, *SD*: 3.1 at baseline and 3.3 at the 50th-minute, ΔUPDRS range = 0.50–7.0) and 0% (ΔUPDRS = 0.20) in the sham stimulation group (mean UPDRS motor scores: 15.25 at baseline, 15.05 at the 50th-minute, *SD*: 5.09 at baseline and 5.15 at the 50th-minute, ΔUPDRS range = -3.5 to 4.0).

One-way repeated measures ANOVA was also performed for the difference in each group’s baseline and post-stimulation UPDRS scores (ΔUPDRS comparison). The ΔUPDRS comparison for the active vs. dry needling groups and the active vs. sham groups were significant (*p* < 0.001 for both comparisons); however, the comparison of ΔUPDRS for the sham vs. dry needling groups was not statistically significant (*p* = 0.319).

**FIGURE 8:**
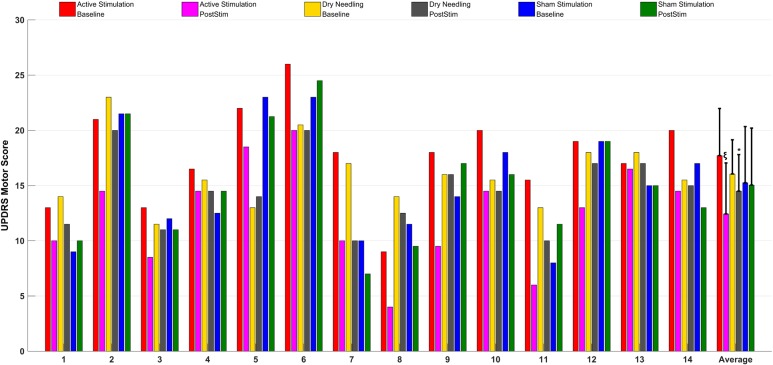
The UPDRS part III scores at baseline and at the 50th-minute for the active group (electrostimulation of the IAMZ), IAMZ dry needling group, and sham stimulation group and overall comparisons of the second arm. ^∗^Statistically significant, ξstatistically and clinically significant.

**FIGURE 9:**
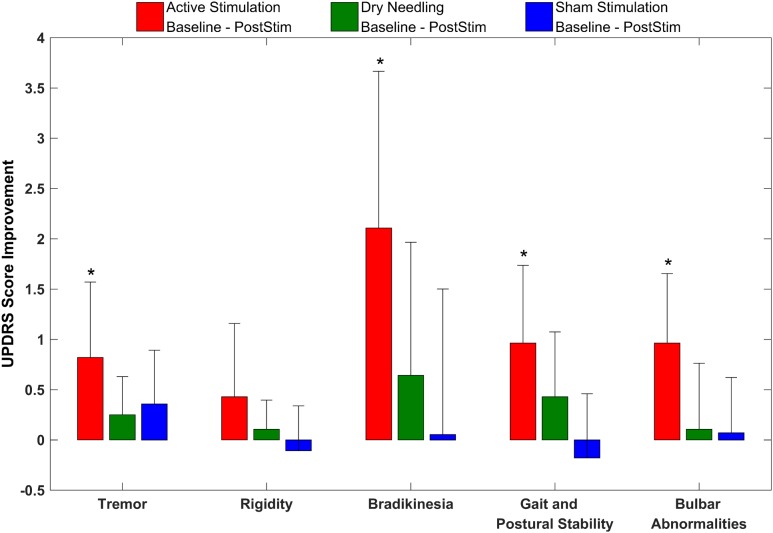
The classified UPDRS subscore differences for group comparisons with Bonferroni correction of the second arm of the study. ^∗^Statistically significant.

**Figure 8** gives the UPDRS Part-III scores and the group comparison for each case and the overall summary of the second arm. **Figure 9** summarizes the classified UPDRS subscore differences in the group comparisons with Bonferroni correction. **Table 4** summarizes the *p*-values for *t*-tests and after Bonferroni correction of the classified UPDRS subscores for the 50th-minute compared with baseline.

**Table 4:** The *p*-values after Bonferroni correction of the classified UPDRS subscores for the 50th-minute compared with baseline of the second arm study groups.

	*p*-Values - Baseline vs. Post-stim		
Subgroup	Active	Dry needling	Sham
Tremor	***0.001***	*0.029*	*0.027*
Rigidity	*0.047*	0.189	0.385
Bradykinesia	***<0.001***	0.092	0.892
Gait and postural instability	***<0.001***	*0.028*	0.315
Bulbar abnormalities	***<0.001***	0.551	0.635

**t*-test; *p* < 0.05, Bonferroni correction *p* < 0.016. Italic: significant in *t*-test, bold: significant after Bonferroni correction, bold and italic: significant in *t*-test and Bonferroni.*

Bilateral assessments of the symptomatic improvements of PD patients before and after electrical stimulation of the IAMZ revealed that unilateral stimulation of the intrinsic auricular muscles can relieve PD symptoms on both sides at the 50th-minute. The 50th-minute mean improvements in the lateralized (UPDRS Part III subscore 20–26) UPDRS motor subscores were 2.39 on the contralateral side and 1.89 on the ipsilateral side in the active group. The 50th-minute differences in the contralateral and ipsilateral UPDRS motor subscores were 0.79 and 0.61 in the dry needling group and 0.48 and 0.0 in the sham group, respectively.

## Discussion

The results of the present study demonstrated a substantial improvement in UPDRS motor scores after electrostimulation of the IAMZ but not after placebo or sham stimulation. The UPDRS score improvements in the electrostimulation of IAMZ groups were statistically and clinically significant. Comparison of the ΔUPDRS (5.9 in the first arm and 5.29 in the second arm) of the active groups considering the clinically important difference (CID) values described by Shulman et al. (ΔUPDRS 2.5, 5.2, and 10.8 for minimal, moderate, and large CIDs, respectively) indicated that the improvement in UPDRS scores obtained with electrostimulation of the IAMZ in the present study falls between a moderate and large CID (Shulman et al., 2010). In addition, dry needling of IAMZ group reached to a statistically significant improvement (but not a CID) in UPDRS scores which was not the case for the sham or placebo groups. The dry needling group results also underlined the significance of the IAMZ. Invasive deep brain stimulation modalities of neuromodulation utilize bilateral approaches to document UPDRS improvement rates. It must be noted that the results of the present study were obtained using only unilateral stimulation, and bilateral stimulation needs to be investigated.

In addition to UPDRS score improvements, the classified UPDRS subitem paired *t*-test analysis also demonstrated that symptomatic suppression for all subitem groups in both arms of the study, including tremor, rigidity, bradykinesia, gait and postural stability and bulbar abnormalities, were statistically significant for only the electrostimulation of IAMZ group. All of the other study groups were not statistically significant after *t*-test analysis for all of the subitem analysis.

The Bonferroni correction is a conservative approach that has advantages and disadvantages, especially for pilot studies. To clarify the outcomes of the conservative Bonferroni correction, we also performed analyses of the subscore groups. The suppression of tremor, rigidity, gait and postural stability and bradykinesia symptoms were still statistically significant after Bonferroni correction in the first arm of the study, and all subgroups (except rigidity) exhibited statistically significant reductions after bonferroni correction in the second arm of the study. In addition, although the improvements in bulbar abnormalities of the first arm and rigidity in the second arm were not statistically significant after the Bonferroni correction, there was a trend toward significance in both groups (p-crit: 0.025, *p*: 0.030 for bulbar abnormalities in arm 1; and p-crit: 0.016, *p*: 0.047 for rigidity in arm 2). Axial symptoms, including speech, have also been reported to worsen with DBS-STN (Tornqvist et al., 2005; Hammer et al., 2010; Moreau et al., 2011; Sidiropoulos et al., 2013). None of the subitems worsened in the active stimulation group in the present study, in contrast to other DBS-STN applications (Tornqvist et al., 2005; Hammer et al., 2010; Moreau et al., 2011; Sidiropoulos et al., 2013). On the other hand, DBS-PPN has been reported to induce greater improvements in PIGD (Hamani et al., 2007; Tykocki et al., 2011). In the context of the different outcomes of DBS-STN and DBS-PPN on motor symptoms and the outcomes in the present study, we suggest that the underlying mechanism of IAMZ stimulation may be related to PPN-associated pathways rather than STN-associated pathways or that both the STN and the PPN may be responsible for the observed effect; however, a sole contribution of the STN is not suggested.

The results of the bilateral assessments of the symptomatic improvements of PD patients before and after electrical stimulation of the IAMZ revealed that unilateral stimulation (ipsilateral to dominant, lateralized motor symptoms) of the IAMZ relieved PD symptoms on both sides of patients in both arms of this study. These improved contralateral motor symptoms may suggest a bihemispheric contribution of the intrinsic auricular muscles. Whether the stimulation of facial nerve branches over the intrinsic auricular muscles is solely responsible for all of the bilateral symptomatic improvements observed in this study or whether other nerves in the IAMZ also contribute remains unclear. The C2 spinal nerve may also play a role in bilateral symptomatic improvement. Although the C2-related role of the propriospinal tracts is more active on the ipsilateral side, animal studies demonstrated that C2 spinal nerves have ipsilateral and contralateral connections with the interpositus nucleus of the cerebellum (Matsushita and Xiong, 2001). As such, C2 spinal nerve stimulation may act on the bilateral PPN through bilateral cerebellar nuclei, and bilateral PPN stimulation may act on the bilateral reticulospinal tract to modulate bilateral motor symptoms. However, C2 zone stimulation in the sham group of the second arm was ineffective.

Autonomic nerve modulation may also contribute to the bilateral symptomatic relief observed in the present study. Low-frequency vagal nerve stimulation leads to impaired vagus nerve function and inhibition of the dopamine system in brain structures (Ziomber et al., 2012). Considering that low- and high-frequency electrostimulation have different outcomes on the autonomic nerve system and on neurotransmitters (Cakmak et al., 2008, 2016; Zhao, 2008), high-frequency stimulation of the Arnold branch of the vagus nerve may in fact have the opposite effect. The present study used 130 Hz stimulation, which is very high compared with the frequency (0.5 Hz) that inhibits the dopaminergic system via vagus nerve stimulation (Ziomber et al., 2012). Therefore, the beneficial bilateral effects obtained in the present study may also be derived from improved dopamine levels. Although there was not a formal feedback survey, the patients in the active group also reported that they felt like they had their levodopa pills. The underlying neurotransmitter related mechanism of action will be the focus of animal studies in addition to levodopa integrated clinical trials for the IAMZ stimulation.

A recent study reported bilateral c-fos activation in the nucleus tractus solitarius (NTS) and the locus coerulei (LC) after stimulation of the left cavum concha area, which is located in the bed of the Arnold branch of the vagus nerve in rats (Ay et al., 2015) (**Figures 1A,B**). On the other hand, transganglionic neurotracing studies with horseradish peroxidase (HRP) injections to the central cut end of the Arnold branch did not result in LC labeling (Nomura and Mizuno, 1984). Interestingly, the same study investigated left cavum concha stimulation and reported c-fos activation in the facial nerve nucleus in one of the experimental animals. The cavum concha is located next to the helicis minor muscle, and facial nerve fibers to the helicis minor may also be distributed in this area. Therefore, it is always possible that some facial nerve fibers will be stimulated along with the Arnold branch of the vagus nerve within the cavum concha. The authors attributed the c-fos labeling in the bilateral LC to projections from the NTS and as a result of stimulation of the Arnold branch of the vagus nerve. The Arnold branch of the vagus-NTS-LC axis may not be the only option for bilateral LC c-fos labeling after cavum concha stimulation, as sympathetic nerves also contribute to the cavum concha area within the arterial walls, and the cervical sympathetic ganglia were not investigated for possible labeling in that particular study. A major input to the LC originates from the nucleus paragigantocellularis (nPGi) in the reticular formation (Ennis and Aston-Jones, 1988; Kessel et al., 2008) (**Figure 1B**). The nPGi directly innervates sympathetic neurons and receives inputs (Ennis and Aston-Jones, 1988; Aston-Jones et al., 1996; Berntson et al., 1998) (**Figures 1A,B**). This pathway is involved in transmitting information of sympathetic control and state between the nPGi and the LC (Garcia-Rill, 1991; Limousin et al., 1997) (**Figures 1A,B**). In addition, the nPGi has been shown to be antidromically activated by electrostimulation of the LC, which may also be the case for sympathetic afferents. The major efferent pathways of the LC, the major wakefulness-promoting nucleus, project to the PPN (Samuels and Szabadi, 2008) (**Figure 1B**). Therefore, the possible role of the auricular sympathetic nerves in LC activation and, as a consequence, their possible effects on motor regulation via the LC-PPN axis, cannot be excluded. In contrast, the sham stimulation zone, which included the C2 and sympathetic nerves in the second arm of the study, did not affect the UPDRS motor scores, which may suggest that the sympathetic nerves had a minimal role in the UPDRS improvements observed in the present study. On the other hand, the distribution of the sympathetic nerves may have less contribution to sham zone stimulation area in comparison to the IAMZ electrostimulation zones so that sympathetic nerves may still have a potential role in the observed clinical improvement.

Although there are different possible anatomical pathways from the IAMZ to the LC-PPN axis that need to be investigated, the demonstration of bilateral LC activation through unilateral stimulation of the cavum concha area (Ay et al., 2015) may also explain the bilateral motor improvements observed in our study.

Proprioception nerve fibers of the intrinsic auricular muscles may project to the mesencephalic trigeminal nucleus, to the cuneate nucleus, or both. The mesencephalic trigeminal nucleus projects to the reticular formation and to the spinal cord (Usunoff et al., 1997) (**Figures 1A,B**). This connection indicates the presence of potential intrinsic auricular muscle proprioception feedback over reticular formation, which is the PPN’s gateway for influencing movement and posture. On the other hand, imaging studies have revealed that the cortical representation of the ear is distributed not as a single zone over the cortical face-head representation area but as numerous different zones over the face, head and neck representation areas (Nihashi et al., 2001). If this unique auricular sensory representation finding is extrapolated to intrinsic auricular muscle representations, then the proprioceptive feedback of the intrinsic auricular muscles may also contribute to the neck proprioceptive center, the nucleus cuneatus. A recent study also supported such a connection: in an fMRI study, stimulation of the antitragicus muscle zone as a control demonstrated activation of the nucleus cuneatus, whereas there was no activation in the nucleus cuneatus when a non-muscular area of the ear was stimulated (Frangos et al., 2015) (**Figures 1A,B**). The nucleus cuneatus and the PPN are the two major components of the mesencephalic locomotor region that modulates PGID (Garcia-Rill, 1991). The SCM, where the transcutaneous electrode was placed, may also play a role in nucleus cuneatus modulation because of the alternating anode-cathode stimulation method that we used in the present study. On the other hand, the effect would be relatively small compared with the effect that occurs by IAMZ stimulation because of the transcutaneous electrode usage and the very low power of the stimulus. We did not observe any contractions of the SCM during stimulation. In addition, the patients also reported a distinct tingling sensation over the IAMZ but not of the SCM where the transcutaneous electrode was placed. The different surface size of the needle electrodes placed on the IAMZ compared with the transcutaneous electrodes on the SCM may be the reason behind this difference, in addition to the skin conductance factor between the needle electrodes and the transcutaneous electrodes. In conclusion, the contribution of the SCM electrode to the symptomatic improvements observed in the present study, if any, would be minimal. The sham region in the present study is selected to eliminate the potential effect of stimulation of only the C2/sympathetic nerve by ensuring no or minimal stimulation of the SCM, the vagus facial nerve and the trigeminal nerve, on the other hand the character of the sham design has limitations for surface SCM stimulation contributions (if any). Different sham group designs are needed to eliminate the SCM only stimulation effects.

The upper face muscles, such as the orbicularis oculi and frontalis muscles, have similar bilateral hemispheric connections; however, reports of the muscle spindle content of the orbicularis oculi highlight the lack of muscle spindles in this muscle group (Urban et al., 2004). In addition, these muscles are actively used to as mimics and as sphincters, and the contraction of these muscles with external electrostimulation may induce visible contractions that counteract their functions. In conclusion, these muscles are not the ideal targets, not only because they lack muscle spindles but also because they are actively used in daily life, and therefore, the contraction of these muscles may be annoying to patients. Subthreshold stimulation that does not induce visible contractions of these muscles may be another option for an alternative non-invasive electrostimulation modality for modulating similar networks related to IAMZ, but the lack of muscle spindles is still a major concern. On the other hand, intrinsic auricular muscles have active roles in shaping the external ear surface features during the prenatal period and become functionally regressed but anatomically intact in adult humans, such that stimulation of intrinsic muscles does not induce the visible contractions observed in the frontalis and orbicularis oculi (Zerin et al., 1982; Yotsuyanagi et al., 1999). Finally, although no studies on the muscle spindle content of human intrinsic auricular muscles have been published thus far, muscle spindles have been examined in peri-auricular muscles in non-human primates, including, rhesus monkeys (Lovell et al., 1977). The statistically significant (but clinically not significant) effect that was observed in the IAMZ dry needling group in the second arm of the study also underlines the significance of the IAMZ zone during minimal stimulation. In addition, it is worth noting that electrostimulation with the same parameters of a muscle-free area of the auricula in the sham group had no effect on UPDRS scores.

The current study was a pilot study performed on a small group of patients that investigated the immediate effects of IAMZ stimulation on PD motor symptoms. Although the study includes numerous groups including sham, placebo, dry needling and active, the SCM contribution (if any), and the potential perception differences of the sham and real IAMZ stimulations were the limitations. The results require further validation in subsequent studies with more participants. Further investigations of bilateral stimulation applications and different stimulation frequencies are also needed to clarify the efficacy of the technique, as in DBS, for patient-specific frequency effects on tremor. In addition, although postural tremor and tremor at rest significantly improved after active stimulation, we observed that tremor developed more erratically and was modulated by the emotional states of patients. In addition, the patients also well-tolerated the device without any side effect or discomfort with short term (20 min) stimulation. The usability of the device for longer terms will be the focus of future trials. Responsiveness regarding the stage of the disease, different stimulation frequencies and long-term effects should also be investigated. Moreover, we only observed the short-term effects of stimulation, and the second arm of the study indicates that the effect may persist up to the 30th-minute after termination of stimulation, which corresponded to the 50th-minute after the initiation of the stimulation. Prolonged efficacy will also be the focus of the next phase of clinical trials. Responsiveness regarding the stage of the disease must also be investigated in future studies.

## Conclusion

We provide the first demonstration of a potential role of IAMZ electrical stimulation in improving (moderate to large clinical improvement) the clinical motor symptoms of PD patients in the short term. The underlying neuronal pathways and mechanisms need to be investigated in future studies.

## Author Contributions

Developed the concept: YC; experiment design: YC, YO, SE; performed stimulation experiments: YC, BO; performed patient enrollments and/or clinical assessments: HA, GK, SE, AG, OC; development of hardware: HU, SO, YC; development of software and code writing: HU, SO, BO, YC; development of analytical tools: HU, SO, BO, YC; data collection: BO, YC, OC, SO; analyzed data: YC, SE, AG, BO, YO, SE; wrote the draft: YC; wrote the main paper: YC, HA, GK, SE, AG, BO, OC, YO, HU, BO.

## Conflict of Interest Statement

YC, HU, SO, and BO have a related pending patent application. The other authors declare that the research was conducted in the absence of any commercial or financial relationships that could be construed as a potential conflict of interest.
